# Optimizing Western Flower Thrips Management on French Beans by Combined Use of Beneficials and Imidacloprid

**DOI:** 10.3390/insects6010279

**Published:** 2015-03-23

**Authors:** Johnson O. Nyasani, Sevgan Subramanian, Hans-Michael Poehling, Nguya K. Maniania, Sunday Ekesi, Rainer Meyhöfer

**Affiliations:** 1 Kenya Agricultural and Livestock Research Organization, Embu Research Centre, P O Box 27-60100 Embu, Kenya; E-Mail: Johnson.Nyasani@kalro.org; 2 Institute of Horticultural Production Systems, Section Phytomedicine, Leibniz Universität Hannover, Germany, Herrenhäuser Strasse 2, Hannover, 30419, Germany; E-Mails: poehling@ipp.uni-hannover.de (H.-M.P.); meyhoefer@ipp.uni-hannover.de (R.M.); 3 International Centre of Insect Physiology and Ecology, P O Box 30772-00100 Nairobi, Kenya; E-Mails: jnyasani@icipe.org (J.O.N.); ssubramania@icipe.org (S.S.); nmaniania@icipe.org (N.K.M.); sekesi@icipe.org (S.E.)

**Keywords:** *Amblyseius*, benefit-cost ratio, *Frankliniella occidentalis*, entomopathogenic fungus, neonicotinoid, *Phaseolus vulgaris*, predatory mite

## Abstract

Western flower thrips (WFT), *Frankliniella occidentalis* (Pergande), is an important pest of vegetable crops worldwide and has developed resistance to many insecticides. The predatory mites *Neoseiulus* (=*Amblyseius*) *cucumeris* (Oudemans), the entomopathogenic fungus *Metarhizium anisopliae* (Metsch.), and an insecticide (imidacloprid) were tested for their efficacy to reduce WFT population density and damage to French bean (*Phaseolus vulgaris* L.) pods under field conditions in two planting periods. *Metarhizium anisopliae* was applied as a foliar spray weekly at a rate of one litre spray volume per plot while imidacloprid was applied as a soil drench every two weeks at a rate of two litres of a mixture of water and imidacloprid per m^2^. *Neoseiulus cucumeris* was released every two weeks on plant foliage at a rate of three mites per plant. Single and combined treatment applications reduced WFT population density by at least three times and WFT damage to French bean pods by at least 1.7 times compared with untreated plots. The benefit-cost ratios in management of WFT were profitable with highest returns realized on imidacloprid treated plots. The results indicate that *M. anisopliae*, *N. cucumeris*, and imidacloprid have the potential for use in developing an integrated pest management program against WFT on French beans.

## 1. Introduction

French bean, (*Phaseolus vulgaris* L.) (Fabales: Fabaceae), is the second most important horticultural export crop after cut flowers in Kenya, constituting nearly 24% by volume and value of all fresh horticultural exports [1]. In Kenya, small- and large-scale farmers grow French beans throughout the year. Attacks by various pests and diseases remain a limiting factor for French bean production and marketing [2]. The main pests attacking French beans are thrips, whiteflies, aphids, and the bean fly [2]. However, the western flower thrips (WFT), *Frankliniella occidentalis* (Pergande) (Thysanoptera: Thripidae), is considered to be one of the most important pests affecting French bean production [2,3,4]. Farmers commonly use synthetic chemical pesticides to control pests and diseases on French beans. Nevertheless, WFT is difficult to control with insecticides because of its cryptic feeding habit, ability to pupate in the soil [5], and fast development of resistance to the commonly used synthetic chemical insecticides [6,7]. In the recent past, the stringent measures by the European farm management system related to good agricultural practice (GLOBALGAP, formerly EurepGAP standard) on allowed maximum residue levels (MRLs) for banned products, have seen many Kenyan companies and farmers de-listed as a result of interceptions at the export market. In the context of integrated pest management (IPM), cultural, biological, and chemical control have to be judiciously used to provide targeted and efficient pest management solutions that can be tailored to specific climates and habitats [8]. Biological control could be a major component of IPM programs of WFT [9].

Among natural enemies, the predatory mite, *Neoseiulus* (=*Amblyseius*) *cucumeris* (Oudemans) (Acari: Phytoseiidae), is an effective biological control agent for controlling WFT on greenhouse vegetable crops worldwide [10,11]. The efficacy of the entomopathogenic fungus, *Metarhizium anisopliae* (Metsch.) (Hypocreales: Clavicipitaceae), against WFT has been demonstrated on French beans [12] in field cages, and chrysanthemum [9] under greenhouse conditions. Imidacloprid has been reported to be effective in the management of WFT on greenhouse eggplant [13] when applied as a soil drench. However, limited studies have been published on the use of *M. anisopliae*, *N. cucumeris,* and imidacloprid, in management of WFT on French beans under field conditions. Therefore, the aim of this study was to evaluate the potential of combining different control agents such as biological (*M. anisopliae* and *N. cucumeris*) and an insecticide imidacloprid at recommended application rates in the management of WFT on French beans.

## 2. Materials and Methods

### 2.1. Experimental Site

Experiments were carried out at the Kenya Agricultural and Livestock Research Organization (KALRO), Embu Research Centre, Kenya, in two planting periods: March to June and June to September 2011. KALRO-Embu station is characterized by its fairly flat and structurally good landscape. The station is located in the Upper Midlands 2 (UM2) agro-ecological zone at an altitude of 1480 m (0.501291° S, 37.458664° E). Its soils are classified as Humic Nitisols. It receives a mean annual rainfall of 1238 mm. The rainfall is bimodal, with first rains starting from late March to May and second rains starting from October to December [14].

### 2.2. Insect

Initial cultures of WFT were field-collected from French beans at Thika, Kenya, in January 2011. Sample specimens were further processed in the laboratory to confirm the identity of the thrips using LucID key [15,16]. The WFT were reared in ventilated plastic jars (9 × 15 × 8 cm). A hole was cut on the lid and covered with thrips-proof organdy cloth (mesh openings of 95 μm) to allow ventilation. The thrips were reared at the International Centre of Insect Physiology and Ecology’s insectary at 25 °C ± 1 °C, 50%–60% relative humidity (RH) with a 12 h L:12 h D photoperiod as described by Nyasani *et al.* [4]. French bean pods surface-coated with sugar and honey solution were used as a food supplement. Forty (40) female and 10 male adult WFT were aspirated from the collection and transferred into ventilated plastic jars provided with 4 pieces (8–10 cm length) of French bean pods. After 3 days, the egg infested bean pods were transferred to fresh ventilated plastic jars described earlier. First instar larvae (L1) started to hatch 2 days later. Three days post-eclosion second instar larvae (L2) emerged. The L2 were carefully brushed off the French bean pods into a new jar with fresh pods and tissue paper at the base for pupation. Five days later newly hatched adults emerged. Forty (40) females and 10 males of newly hatched adult WFT were sequentially transferred to new ventilated plastic jars provided with French bean pods as described earlier. The fourth and seventh generations of thrips from these cultures were used in the March–June and June–September experiments, respectively. To determine the sex of newly hatched thrips (2 days old), 5 thrips were placed in a 6-cm diameter Petri dish and classified under the stereomicroscope at 40× magnification based on external morphological differences. Adult female and male WFT were aspirated separately and transferred into individual ventilated plastic jars. At the onset of budding, which is the critical stage for infestation by thrips [3,17], the newly hatched adult WFT (3 days old) were released using a modified straw onto the middle leaves of French beans with 5 adult thrips (1:4, male:female) per plant and left to acclimatize for one day. A total of 616 plants per plot were infested with WFT representing 76% of the plants in each experimental plot. The releases were made between 06:00 and 08:00 hours when the thrips are known to be inactive.

### 2.3. Crop

*Phaseolus vulgaris* (Cv. Serengeti) seeds were planted in the experimental plots measuring 8 × 4.5 m (length × width) at 50 × 10 cm spacing (rows × plants) on a flat bed in an open field. At planting, 10 g of di-ammonium phosphate (DAP) fertilizer were applied per hill. Two French bean seeds were planted per hill and later thinned to one per hill. A randomized block design was adopted with four replicates per treatment. There were 5 m buffer zones left between the experimental plots and blocks. The buffers were kept weed-free throughout the experimental periods to avoid re-infestation of French bean plants by thrips from the surrounding plots. In addition, the surroundings of the experimental fields (30 m all round) were kept weed-free before and during the experiments to prevent infestation from the surroundings. No fungicide, foliar feed, or insecticide (other than imidacloprid) was applied on the French bean plants during the entire experimental periods. Cultural practices as recommended by the Ministry of Agriculture were adopted [14].

### 2.4. Fungus

The entomopathogenic fungus used in the present study was Campaign^®^ (*Metarhizium anisopliae* isolate ICIPE 69) and was obtained from the Real IPM Company (K) Ltd, Thika, Kenya. It was isolated in 1990 in the Democratic Republic of Congo and was selected because of its efficacy against WFT [9] and bean flower thrips, *Megalurothrips sjostedti* (Trybom) [18] and its availability in Kenya. The fungus was stored in a refrigerator at 5 °C following the supplier’s recommendation. The spore count was 10 × 10^11^ colony forming units (CFU) per mL at the time of purchase.

### 2.5. Predatory Mite

The predatory mite used in the present study was *Neoseiulus cucumeris* and was obtained from the Real IPM Company (K) Ltd, Thika, Kenya. The predatory mite was pre-mixed with wheat bran and a prey mite *Tyrophagus tropicus* Robertson at source. To determine the sex ratio of the mass-reared predatory mites, 200 adult mites were taken from each container and observed under a binocular microscope at 40× magnification. The mites were classified as male or female based on external morphology as described by Kim *et al.* [19]. The sex ratio was 1:1 (female to male). To determine the quantity of live mites at each field release, the number of live mites in ten 0.1 g samples of the product was counted after gently rotating the bottle containing the mites to ensure evenly mixed material. The survival rate of the mites was 100%.

### 2.6. Insecticide

Imidacloprid (Confidor 70 WG, imidacloprid 700 g/kg) was procured from a pesticide dealer and selected because it was among the commonly used insecticides by Kenyan French bean farmers during a baseline survey on the seasonality of WFT [17].

### 2.7. Treatments

Six treatments were adopted in this study: (1) untreated plots; (2) plots receiving *N. cucumeris* releases only; (3) plots drenched with a synthetic insecticide, imidacloprid; (4) plots with plants sprayed with entomopathogenic fungus, *M. anisopliae*; (5) plots with plants drenched with imidacloprid and receiving *N. cucumeris* releases; and (6) plots with plants sprayed with *M. anisopliae* and receiving *N. cucumeris* releases. The effect of combined application of imidacloprid and *M. anisopliae* (ICIPE 69) was not tested because prior laboratory experiments indicated that the combination did not result in synergistic effects on WFT [20]. One trial with the six treatments in each was conducted from March–June and a second from June–September 2011. Each treatment was replicated four times in plots measuring 8 × 4.5 m (length × width) in a randomized block design. The same design was used in the two trials. The first application of imidacloprid and *M. anisopliae*, and release of *N. cucumeris* was at the budding stage of French beans, one day after artificial release of WFT. Thereafter, specific treatments were applied either weekly or after every second week. *Metarhizium anisopliae* was applied weekly for six weeks as a foliar spray at a rate of 1 litre spray volume per plot (36 m^2^) (1 mL of *M. anisopliae* (1 × 10^11^ colony forming units (CFU) mL^−1^) per 1 litre of water). *Neoseiulus cucumeris* was released on plant foliage once every 14 days (with four applications per planting period) at a rate of 3 mites/plant or 57 mites/m^2^/release after soil application of imidacloprid and spraying the French bean plants with *M. anisopliae* in respective treatments. Only adult mites (2 days old) were used in the experiments. To optimize the release rates of the predatory mites, toothpicks were tipped into the containers carrying mites to allow them to climb on. When three adult mites were observed on the toothpick with help of a magnifying glass, the toothpick was gently placed on the middle leaves of each French bean plant in selected treatments. Imidacloprid was applied once every 14 days (with three applications per planting period) as a soil drench at a rate of 2 litres of mixture per m^2^ (10g product per 20 litres water). Imidacloprid and *M*. *anisopliae* were applied using a knapsack sprayer and no adjuvant was used during application. Application rates of all treatments were as per suppliers’ recommendations.

### 2.8. Evaluation of Treatments

Sampling for thrips through whole plant tapping method [21] was conducted one day after treatment application to establish a baseline for future comparisons and then switched back to weekly sampling till crop senescence. In each experimental plot 20 individual French bean plants were shaken over a white enamel tray for 15 seconds. Plants sampled for thrips were tagged so as to allow other plants to be sampled in the next field observation and to avoid sampling some plants more than once. The different developmental stages of thrips collected on the tray were counted and collected in Eppendorf tubes containing 95% ethyl alcohol. At flowering stage, 30 fully opened French bean flowers (3 flowers per plant from 10 plants) from each experimental plot were randomly collected in 20 ml universal bottles containing 95% ethyl alcohol and later transferred to 70% alcohol in the laboratory. Some specimens were retained in 95% alcohol for future molecular analysis. Adult and larval thrips were extracted in the laboratory from the flowers and further processed for identification using the LucID key [15,16].

At podding stage, damage to French bean pods due to WFT feeding was assessed by visual examination. French bean pods from 10 French bean plants from each experimental plot were sampled at random and observed for thrips feeding symptoms. Thrips damage was rated on a scale of 1–5 adopted from McKenzie *et al.* [22] where 1 = no damage (0%), 2 = slight damage (≤25%), 3 = moderate damage (> 25 ≤ 50%), 4 = severe damage (> 50 ≤ 75%), and 5 = very severe damage (>75%). French bean pods within damage scores of 3–5 were weighed to calculate the percentage of unmarketable pods by weight. Yield loss due to thrips feeding was calculated as a proportion of weight of unmarketable French bean pods out of the total yield realized for each treatment.

### 2.9. Statistical Analyses

All count data on WFT were checked for normality and homogeneity of variance using Shapiro-Wilk and Levene tests [23,24], respectively, before analysis. To avoid pseudo-replication, the observed insect counts recorded per plant were converted into additive components and the mean population density per plant was calculated. The weekly count data were subjected to repeated measures analysis of variance (RM-ANOVA). The yield data and damage scores were subjected to analysis of variance (ANOVA) using a generalized linear model (GLM) and package stats [23]. If ANOVA revealed significant differences, pair-wise treatment differences were tested using Tukey’s Honestly Significant Difference (HSD) test. All statistical analyses were performed using R version 2.15.3 [23] at a significance level of 0.05. The benefit-cost analysis (BCA) was calculated based on market prices to assess profitability of each treatment application in reducing WFT population density and WFT damage to French bean pods. All benefits (positives) and costs (negatives) were quantified into a common currency (USD) and their ratio or difference (net benefits) calculated to determine whether the benefits outweigh the costs [25]. Treatment applications with positive net benefits (benefit/cost ratios > 1) were regarded as profitable [25].

## 3. Results

### 3.1. Western Flower Thrips Population Densities over Time by Plant Tapping

#### 3.1.1. Adult Western Flower Thrips

**Figure 1:**
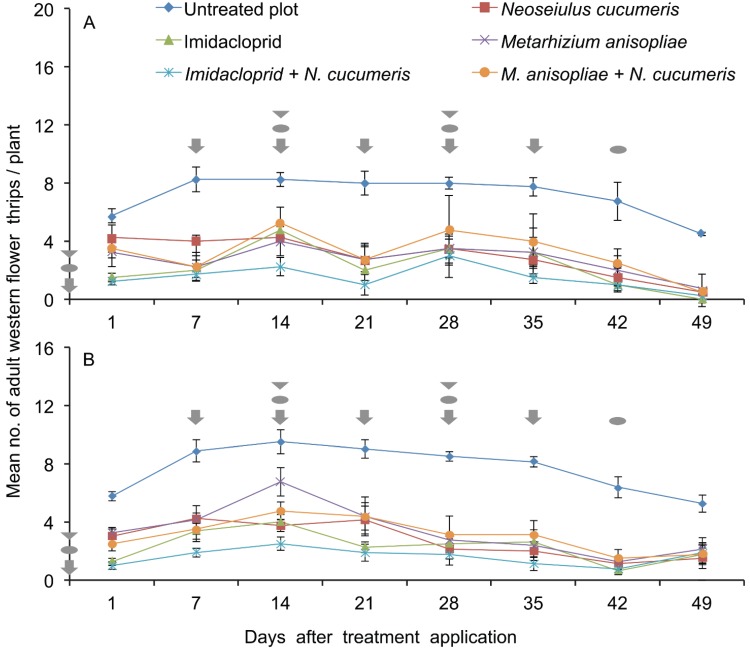
Mean (± SE) number of adult western flower thrips per plant recorded on field-grown French beans in the various treatments over time by plant tapping in March to June (**A**) and June to September (**B**) 2011 at KALRO-Embu, Kenya. Where 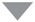 = application of imidacloprid in respective treatments; 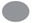 = release of *Neoseiulus cucumeris* in respective treatments; and 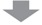 = application of *Metarhizium anisopliae* in respective treatments.

In the first planting period the interaction between treatment and sampling time on the number of adult WFT recorded on French bean plants was not significant by RM-ANOVA (F_35,144_ = 0.636, *p* = 0.940). However, the population densities of adult WFT recorded on French bean plants showed significant differences with various treatments (treatment: F_5,144_ = 44.596, *p* < 0.001) and time (F_7,144_ = 11.196, *p* < 0.001). There was an increase in adult WFT population density from 1 to 14 days (Figure 1A) followed by a gradual decline until 49 days (Figure 1A) in the untreated plots. The thrips densities in untreated plot were always higher than other treatments. All the treatments exhibited fluctuating population densities of adult WFT from day 1–49 according to the treatment applied (Figure 1A).

In the second planting period the interaction between treatment and sampling period on the number of adult WFT recorded on the French bean plants was not significant by RM-ANOVA (F_35,144_ = 1.484, *p* = 0.056). However, the population densities of adult WFT recorded on French bean plants with the various treatments and time showed significant differences (treatment: F_5,144_ = 69.019, *p* < 0.001; time: F_7,144_ = 21.049, *p* < 0.001). In the untreated plots, there was an increase in adult WFT population density from day 1–14 followed by a gradual decline until 49 days (Figure 1B) while in the other treatments the densities of adult WFT fluctuated from day 1–49 although the number of thrips varied according to treatments at each sampling time (Figure 1B). In general, the population densities of adult WFT in untreated plot were always higher than in other treatments. All the other treatments exhibited an increase followed by a decline in adult WFT population density over time although the number of thrips varied according to treatments at each sampling time (Figure 1B). In both planting periods, the least number of adult WFT was consistently recorded on plots with combined application of imidacloprid with *N. cucumeris* releases (Figure 1A,B). Combined application of *M. anisopliae* and *N. cucumeris* releases did not further reduce the density of adult WFT compared with sole application of *M. anisopliae* or *N. cucumeris*. In both planting periods, the population density of adult WFT in untreated plots did not show the expected increase in thrips density to a peak.

#### 3.1.2. Western Flower Thrips Larvae

**Figure 2:**
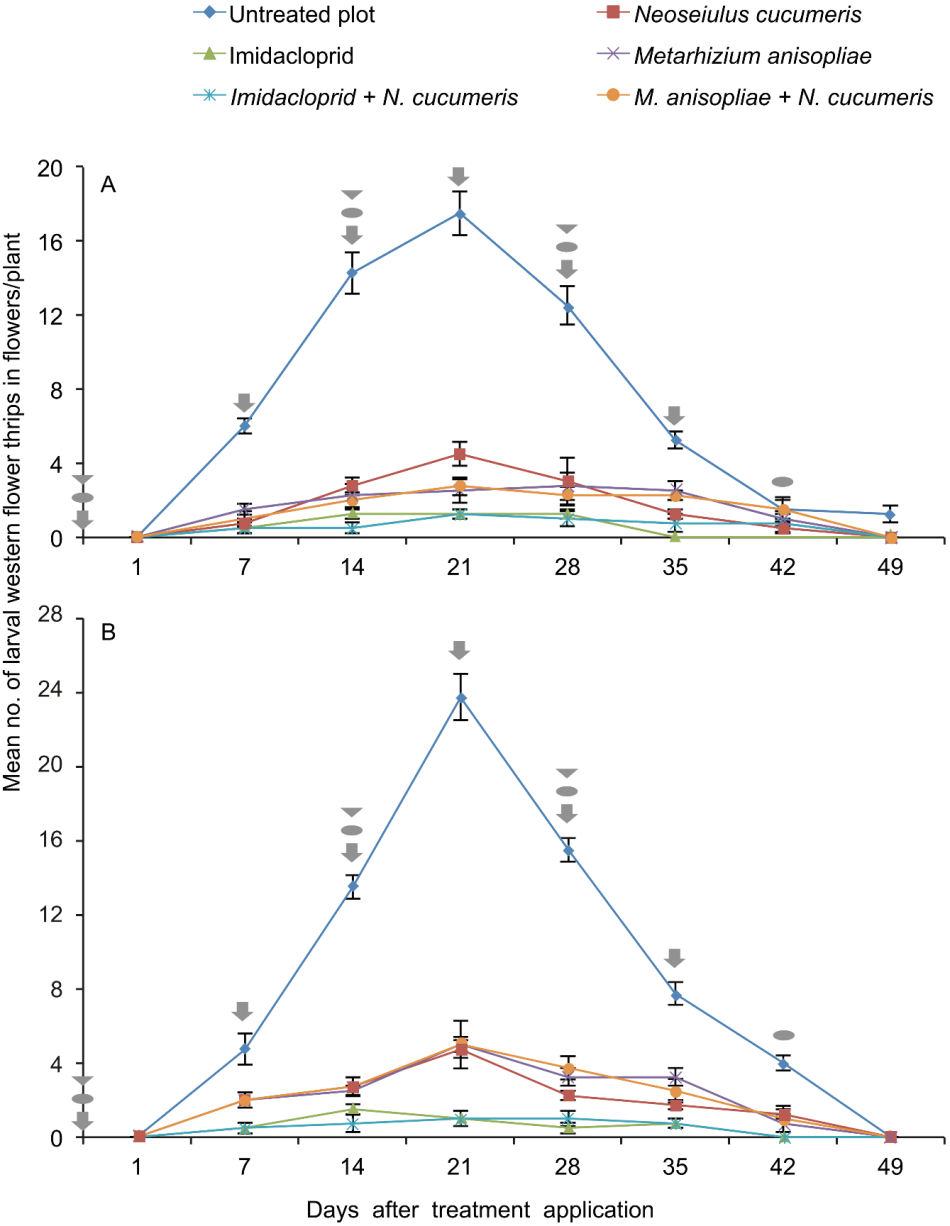
Mean (± SE) number of larval western flower thrips recorded on field-grown French beans in the various treatments over time by plant tapping in March to June (**A**) and June to September (**B**) 2011 at KALRO-Embu, Kenya. Where 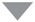 = application of imidacloprid in respective treatments; 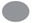 = release of *Neoseiulus cucumeris* in respective treatments; and 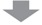 = application of *Metarhizium anisopliae* in respective treatments.

In the first planting period the interaction between treatment and sampling time on the number of WFT larvae recorded on the French bean plants by plant tapping method was significant by RM-ANOVA (F_35,144_ = 24.83, *p* < 0.001). At 21 days from start of sample collection, higher numbers of WFT larvae were recorded in untreated plots compared to those on plots treated with *M. anisopliae,* imidacloprid, and on plots with *N. cucumeris* releases (Figure 2A). The number of larvae recorded in plots treated with sole application of *M. anisopliae* and plots with combined application of *M. anisopliae* with *N. cucumeris* releases were not statistically different 21 days after treatment (2.50 ± 0.6 and 2.75 ± 0.40, respectively) (Figure 2A). Similarly, the number of larvae recorded in plots treated with sole application of imidacloprid and plots with combined application of imidacloprid with *N. cucumeris* releases were not statistically different (1.25 ± 0.25 and 1.25 ± 0.28, respectively) (Figure 2A). The numbers of larvae recorded in plots receiving sole release of *N. cucumeris* were 1.6 times higher compared to those on plots with combined application of *M. anisopliae* with *N. cucumeris* releases 21 days after treatment (Figure 2A).

In the second planting period the interaction between treatment and sampling period on the number of WFT larvae recorded on the French bean plants was significant by RM-ANOVA (F_35,144_ = 43.16, *p* < 0.001). At 21 days from start of sample collection, higher numbers of larvae were recorded in untreated plots compared to those on plots treated with *M. anisopliae,* imidacloprid, and those with *N. cucumeris* releases (Figure 2B). The numbers of WFT larvae recorded in plots with sole release of *N. cucumeris*, plots with sole application of *M. anisopliae* and plots with combined application of *M. anisopliae* with *N. cucumeris* releases were not statistically different (2.75 ± 0.28, 2.50 ± 0.47, and 2.75 ± 0.47, respectively) 21 days after treatment (Figure 2B). Similarly, the number of larvae recorded in plots treated with sole application of imidacloprid and plots with combined application of imidacloprid with *N. cucumeris* releases were not statistically different (1.5 ± 0.28 and 0.75 ± 0.47, respectively). In general, the population density of larvae in the untreated plots increased steadily from day 1–21 followed by a decline from day 21–49 in both planting periods (Figure 2A,B). In both planting periods, with the exception of untreated plots the number of larvae recorded in the other treatments was below five thrips per plant (Figure 2A,B).

### 3.2. Western Flower Thrips Population Densities in Flowers

#### 3.2.1. Adult Western Flower Thrips

**Figure 3:**
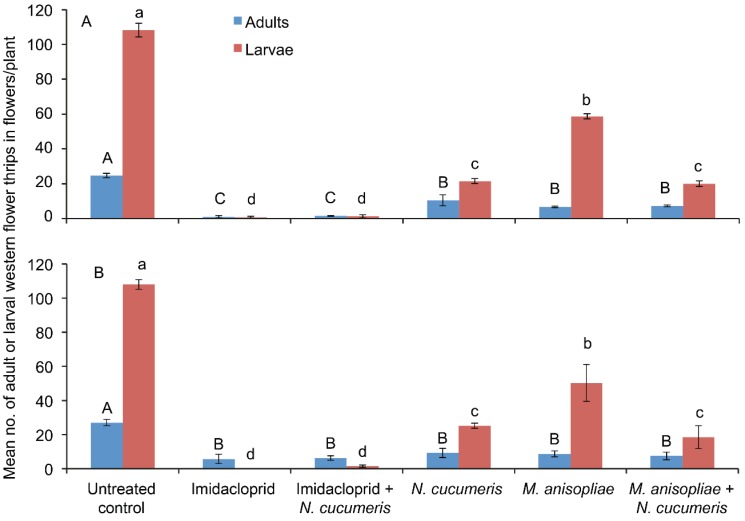
Mean (± SE) number of larval and adult western flower thrips recorded in flowers of field-grown French beans in various treatments in March–June (**A**) and June–September (**B**) 2011 at KALRO-Embu, Kenya. Data are for 21 days after treatment application. Means followed by the same letter in each category indicate no significant differences in the population density of thrips between treatments by *post hoc* comparisons using Tukey’s HSD test: *p* < 0.05.

Since the economic importance of insect herbivores depends greatly on the life stage and tissue of the plant they attack and on their method of feeding, we focused on the flowering stage of French beans, which is the most susceptible stage of the crop to damage by WFT [4,17]. Data on WFT population densities in French bean flowers was collected from the third week after application of treatments. In the first planting period, the effect of treatment on the number of adult WFT recorded on the French bean flowers was significant (F_5,18_ = 10.63, *p* < 0.001). The number of adults recorded on plots treated with sole application of imidacloprid and combined application of imidacloprid with *N. cucumeris* releases were not statistically different (1 ± 0.70 and 1.5 ± 0.28, respectively) (Figure 3A). Similarly, the numbers of adults recorded on plots treated with sole application of *M. anisopliae*, plots with combined application of *M. anisopliae* with *N. cucumeris* releases, and plots with sole release of *N. cucumeris* were not statistically different (6.75 ± 0.47 and 7.25 ± 0.47, respectively). The highest numbers of adult WFT were recorded on untreated plots (Figure 3A).

In the second planting period, the effect of treatment on the number of adult WFT recorded on the French bean flowers was significant (F_5,18_ = 65.75, *p* < 0.001). The numbers of adult WFT recorded on plots treated with sole application of *M. anisopliae*, plots with combined application of *M. anisopliae* with *N. cucumeris* releases, plots with sole release of *N. cucumeris* and plots with sole and combined application of imidacloprid with *N. cucumeris* releases were not statistically different (8.75 ± 1.75, 7.5 ± 2.21, 9.25 ± 2.65 and 6.25 ± 1.25, respectively) (Figure 3B). The highest numbers of adult WFT per flower were recorded on untreated plots (Figure 3B).

#### 3.2.2. Western Flower Thrips Larvae

In the first planting period, the effect of treatment on the number of WFT larvae recorded on the French bean flowers was significant (F_5,18_ = 444.8, *p* < 0.001). The highest numbers of WFT larvae were recorded on untreated plots (Figure 3A). The number of WFT larvae recorded on plots treated with sole application of imidacloprid and combined application of imidacloprid with *N. cucumeris* were not statistically different (0.75 ± 0.47 and 1.25 ± 0.94, respectively) (Figure 3A). Similarly, the number of WFT larvae recorded on plots receiving sole release of *N. cucumeris* and those with combined application of *M. anisopliae* with *N. cucumeris* releases were not statistically different (21.5 ± 1.44 and 20 ± 1.58, respectively). The numbers of WFT larvae recorded on plots treated with sole application of *M. anisopliae* were higher than those recorded on plots treated with imidacoprid and those receiving *N. cucumeris* releases (Figure 3A).

In the second planting period, the effect of treatment on the number of WFT larvae recorded on the French bean flowers was significant (F_5,18_ = 57.5, *p* < 0.001). The highest numbers of WFT larvae per flower were recorded on untreated plots (Figure 3B). The numbers of WFT larvae recorded on plots treated with sole application of imidacloprid and combined application of imidacloprid with *N. cucumeris* were not statistically different (0 and 1.5 ± 0.64, respectively) (Figure 3B). Similarly, the numbers of WFT larvae recorded on plots with sole release of *N. cucumeris* and those with combined application of *M. anisopliae* with *N. cucumeris* releases were not statistically different (25.5 ± 1.49 and 18.5 ± 6.73, respectively). The numbers of WFT larvae recorded on plots treated with sole application of *M. anisopliae* were higher than those recorded on plots treated with imidacoprid and those with *N. cucumeris* releases (Figure 3B).

### 3.3. Pod Yield and Yield Losses due to Thrips Feeding

In both planting periods, there were significant differences in the thrips damage recorded on French bean pods in the various treatments (first planting period: F_5,15_ = 27.33, *p* < 0.001; second planting period: F_5,15_ = 15.22, *p* < 0.001). In both planting periods, the least thrips damage scores (1.25–1.5) were recorded on plots treated with either single application of imidacloprid or combined application of imidaclorid with *N. cucumeris* releases (Table 1). Plots treated with *M. anisopliae* only and plots with *N. cucumeris* releases alone recorded a damage score of 2.25 and 2.5 in the first and second planting periods, respectively. The highest thrips damage score (4) was recorded in the untreated plots in the first and second planting periods (Table 1). The least percentage yield loss (4%–6%) due to WFT feeding was recorded in plots treated with imidacloprid. Plots treated with sole application of *M. anisopliae* and combined application of *M. anisopliae* with *N. cucumeris* releases recorded yield losses of 25%–30% and 23%–25%, respectively. Plots with *N. cucumeris* releases recorded a yield loss of 46%–50%. The highest yield loss of 70%–75% was recorded on untreated plots (Table 1).

In both planting periods, there were significant differences in the pod yield recorded in the various treatments (first planting period: F_5,15_ = 9.66, *p* < 0.001; second planting period: F_5,15_ = 9.60, *p* < 0.001). The highest yield was recorded in the imidacloprid-treated plots while the lowest was recorded in untreated plots (Table 1). In the first planting period, single and combined treatments with *M. anisopliae* and *N. cucumeris* recorded a two times lower yield compared to plots treated with imidacloprid alone (Table 1). However, in the second planting, the pod yield recorded in plots treated with imidacloprid alone was not significantly different from plots treated with imidacloprid and receiving *N. cucumeris* releases, and plots treated with *M. anisopliae* and receiving *N. cucumeris* releases. Compared to plots treated with imidacloprid alone, plots treated with single treatments of *M. anisopliae* and *N. cucumeris* recorded a 1.3 times lower pod yield.

The benefit-cost ratios (BCR) obtained varied according to treatments in the two planting periods (Table 1). All the costs incurred in application of either *M. anisopliae* or imidacloprid and release of *N. cucumeris* for management of WFT are listed in Table 2. The results indicated that all treatment applications for management of WFT on French beans were profitable (BCR > 1). The highest returns in the first (BCR = 2.98) and second (BCR = 3.49) planting periods were obtained from French bean plots treated with imidacloprid alone. However, there were only marginal differences in the returns between application of either imidacloprid or *M. anisopliae* in the first (BCR = 2.98 *vs.* CBR = 2.62) and second (BCR = 3.49 *vs.* BCR = 3.47) planting period. The least benefit-cost ratios were obtained from French bean plots treated with combined application of *M. anisopliae* with *N. cucumeris* releases in the first (BCR = 1.06) and second (BCR = 1.93) planting period (Table 1).

**Table 1:** Effect of thrips on mean (± SE) French bean pod yield, yield loss, and percentage yield loss as influenced by the various treatments at KALRO-Embu, Kenya in 2011.

	Planting I (March–June 2011)				Planting II (June–September 2011)			
Treatment	Pod Yield (kg/ha)	Yield Loss due to Thrips Damage [kg/ha (%)]	Damage Score due to Thrips Feeding	Benefit-Cost Ratio	Pod Yield (kg/ha)	Yield Loss due to Thrips Damage [kg/ha (%)]	Damage Score due to Thrips Feeding	Benefit-cost ratio
Untreated plot	2023.8 + 178.0 c	1416.6 (70)	4.00 a	-	2809.5 + 177.7 c	2017.1 (75)	4.25 a	
*Neoseiulus cucumeris*	2500.0 + 218.8 cb	1250.0 (50)	2.25 b	1.38	4214.3 + 266.3 b	1938.5 (46)	2.75 b	2.51
*Metarhizium anisopliae*	3035.7 + 276.0 cb	758.9 (25)	2.25 b	2.62	4297.6 + 272.4 b	1289.2 (30)	2.50 cb	3.47
*M. anisopliae* + *N. cucumeris*	2440.5 + 213.9 cb	561.3 (23)	1.75 cb	1.06	4571.4 + 287.7 ab	1142.8 (25)	2.25 cbd	1.93
Imidacloprid	4785.7 + 418.6 a	239.2 (5)	1.25 c	2.98	5785.7 + 364.2 a	462.8 (8)	1.25 d	3.49
Imidacloprid + *N. cucumeris*	3690.5 + 324.8 b	147.6 (4)	1.25 c	1.46	5595.2 + 352.1 ab	335.7 (6)	1.50 d	2.16
F_5,15_	9.66		27.33		9.60		15.22	
*P*-value	0.0003		<0.001		0.0003		<0.001	

^a^ Means in a column followed by the same letter are not significantly different (Tukey’s HSD test: *p* < 0.05). ^b^ Damage score scales are as follows: 1 = no damage (0%); 2 = slight damage (≤25%); 3 = moderate damage (>25 ≤ 50%); 4 = severe damage (> 50 ≤ 75%) and 5 = very severe damage (>75%). ^c^ Benefit-cost ratios greater than one (>1) are profitable.

**Table 2:** Costs of insecticide, predatory mites, entomopathogenic fungi, and their application.

Item	Cost Description	Cost (USD/ha/Planting Period; 1 US$ = 80 KES)
1	Cost of insecticide (imidacloprid) used	1042
	Cost of knapsack hire for insecticide application	130
	Cost of spraying insecticide	163
2	Cost of predatory mites (*Neoseiulus cucumeris*) used	577
	Cost of releasing predatory mites	217
3	Cost of entomopathogenic fungi (*Metarhizium anisopliae*) used	174
	Cost of knapsack hire for entomopathogenic fungi application	260
	Cost of spraying entomopathogenic fungi	326
4	Free market price of French beans (1 kg) at the time of the experiment	0.875

## 4. Discussion

Most of the studies on the use of beneficials such as *N. cucumeris* and *M. anisopliae* for management of WFT have been conducted on greenhouse crops. To our knowledge, only a few studies have demonstrated the efficacy of predatory mites and entomopathogenic fungi against thrips in open field experiments [26,27,28]. Therefore, our study contributes to the knowledge on the potential of predatory mites and entomopathogenic fungi in managing WFT under field conditions. Our results indicate that the plant tapping method collected fewer thrips compared with picking of flowers. The differences in thrips numbers by the different sampling methods can be explained by the fact that adult WFT prefer residing in tightly enclosed and concealed spaces of plants [29] over plant foliage, and adult females feed on pollen to ensure stimulated oviposition, reduced larval development time, and increased female fecundity [30,31]. In our field experiments between March and September, the population density of adult WFT did not show the expected increase in thrips density over time in the untreated plots. Contrastingly, previous research has reported that on field-grown French beans, there is a gradual increase in the population density of WFT to a peak and in most cases a decline as the crop enters senescence [17].In our field experiments, the use of imidacloprid alone greatly reduced the numbers of WFT recovered on French bean plants and flowers. Previous research has reported that imidacloprid when applied either as a foliar spray or as a soil drench significantly suppressed both adults and larvae of chilli thrips *Scirtothrips dorsalis* (Hood) on pepper in the field for at least 15 days [32]. However, combined use of imidacloprid with *N. cucumeris* releases did not further reduce the density of WFT on French bean plants. This indicates that *N. cucumeris* is not compatible with imidacloprid. Our results corroborate previous studies that have reported the incompatibility of imidacloprid with *N. cucumeris* [33,34]. The efficacy of *N. cucumeris* against WFT larvae observed in the present study has also been reported against WFT larvae on greenhouse tomatoes [35] and green beans [36]. Our results also indicate that sole application of *M. anisopliae* is more effective in reducing the population density of adult WFT than WFT larvae, which confirms previous studies [9,37]. Combination treatment of *M. anisopliae* and *N. cucumeris* was effective in reducing the population density of both adult and larval WFT in flowers. However, combined application of *M. anisopliae* and *N. cucumeris* releases did not further reduce the density of adult and larval WFT by the plant tapping method. Fungal agents have the potential to negatively affect predators directly/indirectly by infecting them or depleting the prey population [38,39,40]. Similarly, the predators could negatively affect fungal agents by consuming the preys that are infected thereby removing the pathogen [39]. Therefore, further research is needed to determine the compatibility of *M. anisopliae* and *N. cucumeris* with focus on the effect of *M. anisopliae* on feeding activity and behavior of *N. cucumeris*, for example, avoidance of patches of thrips containing infected individuals.

Application of imidacloprid resulted in higher French bean pod yield and lower yield loss due to WFT feeding compared with application of *M. anisopliae* or release of *N. cucumeris*. This could be due to the persistence of the insecticide in nectar, pollen, plant tissues, and soil [41], hence ensuring steady activity against larval and adult WFT. Previous studies have shown that soil-applied imidacloprid protects eggplant (*Solanum* spp) against WFT under greenhouse conditions and results in high yields with low damage to the fruits [13]. Similarly, soil applications of imidacloprid have been reported to reduce the number and duration of probing/feeding bouts by *Frankliniella fusca* on pepper (*Capsicum annuum* L.) and mustard (*Brassica rapa* L.) [42] under greenhouse and field conditions. All beneficials (*M. anisopliae* and *N. cucumeris*) and insecticide (imidacloprid) gave benefit-cost ratios of more than one, indicating cost-effectiveness of the management strategies. Therefore, farmers have the option of selecting from the management options to develop an integrated pest management program against WFT. Moreover, in the two planting periods, *M. anisopliae* and *N. cucumeris* showed consistent results in reducing the population density of WFT and WFT feeding damage to French bean pods. Therefore, the two biocontrol agents could be considered over imidacloprid in developing an integrated management program against WFT since they are safer to applicators, consumers, animals, and the environment. Although imidacloprid was the best in our study, its use has been linked with negative effects on animals [33,34,43], and the environment [44]. There are also cases of development of resistance by WFT to imidacloprid [45] and cases of insecticide residues in the export produce [46]. To ensure effective management of WFT on French beans and other horticultural crops, there is a need to build capacity among smallholder farmers on the appropriate use of WFT management options such as entomopathogenic fungi, predatory mites, and insecticides.

## 5. Conclusions

The results of the present study indicate that *M. anisopliae*, *N. cucumeris,* and imidacloprid have the potential for use as components in developing an integrated pest management program against WFT on French beans. We have also shown that use of beneficials such as *M. anisopliae* and *N. cucumeris*, and an insecticide such as imidacloprid is cost-effective for management of WFT under field conditions. The benefit-cost ratios obtained, though computed for on-station experiments, are likely to be applicable on-farm. We also show that imidacloprid performs reliably well against larval and adult WFT when applied as a soil drench. However, further studies comparing the potential of foliar or soil-application of imidacloprid or *M. anisopliae* in the management of WFT on French beans under field conditions are needed. The residual toxicity of selected insecticides and their effect on natural enemies would need to be determined first. To develop a rotational scheme of beneficials and insecticides, there is a need to study the effect of temporal separation of application and/or release of entomopathogenic fungi, predatory mites, and insecticides in reducing WFT population densities and their feeding damage to French bean pods.
